# Geographical disparities in intentional self-harm hospitalization risks in Florida: a retrospective ecological study

**DOI:** 10.7717/peerj.21783

**Published:** 2026-10-08

**Authors:** Howard Onyuth, Agricola Odoi

**Affiliations:** Biomedical and Diagnostic Sciences, University of Tennessee, Knoxville, TN, United States of America

**Keywords:** Intentional self-harm, Hospitalizations, Geographic disparities, GIS, Geographical information systems, Spatial epidemiology, Tango’s spatial scan statistics, Spatial cluster detection, Spatial empirical bayes smoothing, Florida

## Abstract

**Background:**

Over the last decade, intentional self-harm hospitalization risks have increased in the United States (US). Although there is evidence of geographic disparities in intentional self-harm hospitalization risks, with some areas having significantly higher risks, little is known about these disparities in Florida. Identifying these disparities is useful for guiding prevention programs and tailoring efforts to mitigate the problem. Therefore, the objective of this study was to investigate geographic disparities of intentional self-harm hospitalization risks in Florida.

**Methods:**

Retrospective county-level intentional self-harm hospitalization data for the years 2016–2023 were obtained from Florida Health Charts, a publicly available data dashboard maintained by the Florida Department of Health. County-level age-standardized and spatial empirical Bayesian (SEB) smoothed risks were calculated for four time periods: 2016–2017, 2018–2019, 2020–2021, and 2022–2023. Significantly high-risk clusters were identified using Tango’s flexible spatial scan statistics. Two-sample tests of proportions were used to compare proportions of counties and populations in high-risk clusters between rural and urban areas.

**Results:**

A total of 66,308 intentional self-harm hospitalizations were reported during the study period. Statewide SEB smoothed risks varied geographically, ranging from 25.3 to 1,683.1 hospitalizations per 100,000 persons. The highest risks tended to occur in the northwest, northeast, central, and southwest Florida. A total of four statistically significant high-risk clusters were identified in 2016–2017 (relative risks (RR) = 1.44–15.85), six in 2018–2019 (RR = 1.22–12.79), six in 2020–2021 (RR = 1.21–7.25), and eight in 2022–2023 (RR = 1.20–3.69). The cluster with the highest risks was observed in a single rural northeastern county (Union). Additional high-risk clusters were observed in predominantly urban counties. The proportion of Florida counties included in significant high-risk clusters ranged from 22.4% in 2016–2017 to 38.8% in 2022–2023. Urban counties were significantly more likely than rural counties to be part of high-risk clusters in 2016–2017 (*p* = 0.002) and 2020–2021 (*p* = 0.019), but not in 2018–2019 (*p* = 0.11) or 2022–2023 (*p* = 0.477). The highest proportion of the population in high-risk counties alternated between urban and rural areas, with significantly (*p* < 0.0001) higher urban proportions in 2016–2017 and 2020–2021, and significantly (*p* < 0.0001) higher rural proportions in 2018–2019 and 2022–2023.

**Conclusion:**

Geographic disparities in intentional self-harm hospitalization risks exist in Florida, with high-risk spikes observed in rural counties and widespread risks in urban counties. Study findings are useful for guiding targeted intentional self-harm prevention strategies and needs-based resource allocation geared at reducing/eliminating disparities and improving population health for all.

## Introduction

Intentional self-harm, defined as the deliberate destruction of one’s own body tissue with or without suicidal intent, is an important public health problem. The term can be broadly conceptualized as either non-suicidal self-injury disorder (self-harm without suicidal intent or for purposes not socially sanctioned), or suicidal behavior disorder (self-harm with suicidal attempt) (American Psychiatric Association, 2013; Brager-Larsen, Zeiner & Mehlum, 2024). Intentional self-harm encompasses a heterogenous spectrum of behaviors, with manifestations that vary widely in onset, method, severity, frequency, and underlying intent (Lurigio, Nesi & Meyers, 2024). At the individual level, physical signs often include recurrent cuts, scratches, burns, bruises, bite marks, or other wounds and scars frequently occurring in recognizable patterns, or on easily accessible areas of the body (Nawaz et al., 2021; Oscherwitz et al., 2024). In 2021, there were 5.5 million intentional self-harm incidences globally and this is projected to increase to 10.6 million by 2040 (An et al., 2025). In the United States (US), self-harm affects approximately 5% of adults (>18 years), 15% of college students, and 17% of adolescents (with 13 years being the average age of onset, and females are twice as likely as males to self-harm) (Kerr, Muehlenkamp & Turner, 2010; Muehlenkamp et al., 2012). This problem places a significant burden on the healthcare system resulting in an estimated 334,000 hospitalizations in the US (Illinois Department of Public Health, 2023). Moreover, it is estimated that 15% of all hospitalizations among adults in the US are due to intentional self-harm (Oladunjoye et al., 2020).

The economic burden of the problem in the US is estimated at $27.0 billion ($12.3 billion in medical costs, $3.3 billion in lost work and $11.4 billion in quality of life lost) (Illinois Department of Public Health, 2023). At the individual level, the southeast US ranks in the top quantile for per-patient intentional self-harm costs, with Florida, the most populous state within the region, having an estimated hospitalization cost of $7,000 per patient per week (Gibbs, 2023; Rockett et al., 2023; Peterson et al., 2025). These costs also include the cost of the Florida involuntary Baker Act examination, a legal mechanism that authorizes healthcare personnel to perform an involuntary psychiatric examination and, when indicated, hospitalization of individuals deemed to pose a danger to themselves, including those who self-harm (Gibbs, 2023).

Florida struggles with high numbers of intentional self-harm hospitalizations. For example, a total of 7,316 hospitalizations attributed to intentional self-harm were reported in Florida in 2024 (Florida Department of Health, 2025). Moreover, Florida’s diverse population is characterized by pronounced rural–urban socio-economic disparities that shape mental health outcomes and access/utilization of health services following intentional self-harm (Bigham et al., 2023). Compared to urban areas, rural communities in Florida have been reported to be under-resourced, have the second highest proportion of seniors (*i.e.,* individuals > 65 years old), greater financial insecurity, lower socio-economic status, and increased geographic isolation (National Alliance on Mental Illness, 2021; Nath, 2024). Rural areas in Florida also face shortages of healthcare workforce (including limited availability of mental health and emergency care services) and greater travel distances and time to healthcare facilities, which are disproportionately concentrated in urban centers (Shrader et al., 2023). These structural and socioeconomic disadvantages may increase the risk of intentional self-harm hospitalizations resulting from delayed access to mental healthcare and reduced access to preventative follow-up (Nuako et al., 2022). In contrast, urban areas may have lower barriers to healthcare access and greater availability of behavioral health services, which may facilitate earlier intervention and management of mental health conditions (Shrader et al., 2023). This potentially leads to higher risks of intentional self-harm hospitalizations in socioeconomically disadvantaged communities and lower hospitalization risks in more affluent communities.

There is evidence of geographic disparities in risks of hospitalizations due to intentional self-harm in the US (Fingar & Roemer, 2022; Xi et al., 2025). Among the southeastern states, Florida has the highest risk at 41.4 per 100,000 persons compared to Tennessee at 39.9, South Carolina at 38.7, Mississippi at 35.7, Louisiana at 32.7, and Georgia at 31.5 (Oladunjoye et al., 2020; Fingar & Roemer, 2022). However, while these statewide estimates show the overall burden, they do not capture how this burden is geographically distributed at lower spatial scales such as counties or incorporate formal spatial epidemiologic methods to identify statistically significant clusters within states such as Florida (Onyuth et al., 2025). Therefore, it is possible that these variations exist at sub-state geographical scales (*e.g.*, at the county-level) and if identified may provide useful information to guide health planning, resource allocation and prevention efforts to reduce/eliminate these disparities (Florida Department of Health, 2024). Unfortunately, little has been done to conduct detailed and statistically rigorous investigations of these disparities at the county-level in Florida, and yet this information is important for guiding needs-based resource allocation to address the problem and reduce/eliminate disparities (Rossen et al., 2018; Lee, 2023). This study moves beyond prior descriptive approaches (Swanson et al., 2016; Villa et al., 2023) by applying rigorous spatial analytic methods to provide evidence at lower spatial scale to support targeted prevention and intervention efforts (Onyuth et al., 2025). Therefore, the objective of this study was to investigate county-level geographic disparities in intentional self-harm hospitalization risks in Florida by examining spatial distribution, identifying high-risk clusters, and rural–urban differences in proportion of counties and populations residing in high-risk clusters.

## Materials and Methods

### Ethics review

This study was reviewed by the University of Tennessee Institutional Review Board (IRB) (IRB Number: UTK IRB-25-09098-XM). In accordance with 45 CFR 46.101, the review board determined that the IRB application is eligible for exempt review; informed consent was waived by the IRB. The study used publicly available secondary data aggregated to the county-level. Therefore, the study data could not be used to identify individual participants.

### Study design and setting

This retrospective ecological study was conducted in Florida, the most populous state in southeastern US with an estimated 23.4 million residents and a population density of 401.4 persons per square mile (United States Department of Commerce, 2020; United States Census Bureau, 2025). The 67 counties in Florida are classified as either urban (52.2%) or rural (47.8) (Fig. 1) (United States Department of Commerce, 2020). Based on this classification, rural counties were defined as having populations ≤75,000 or fewer, or a population of 125,000 or fewer that is contiguous to a county with 75,000 or fewer residents (population densities <500 persons per square mile), while urban counties are those that do not meet any these criteria (United States Department of Commerce, 2020). In this study, the classification of a county as either rural or urban was based on the above described county population criteria. This classification approach was selected because it is the classification system used by the Florida Department of Health for health planning, and assessment of geographic disparities across the state (United States Department of Commerce, 2020). Therefore, the findings of this study would be directly applicable to their health planning efforts. The population of the state is 50.6% white (non-Hispanic/Latino), 28.7% Hispanic/Latino, and 16.9% Black or African American (United States Census Bureau, 2025). Approximately 21.6% are aged <18 years, 18.3% are 18–34 years, 13.1% are 35–44 years, 25.2% are 45–64 years and 21.8% are ≥65 years (United States Census Bureau, 2025).

**Figure 1:**
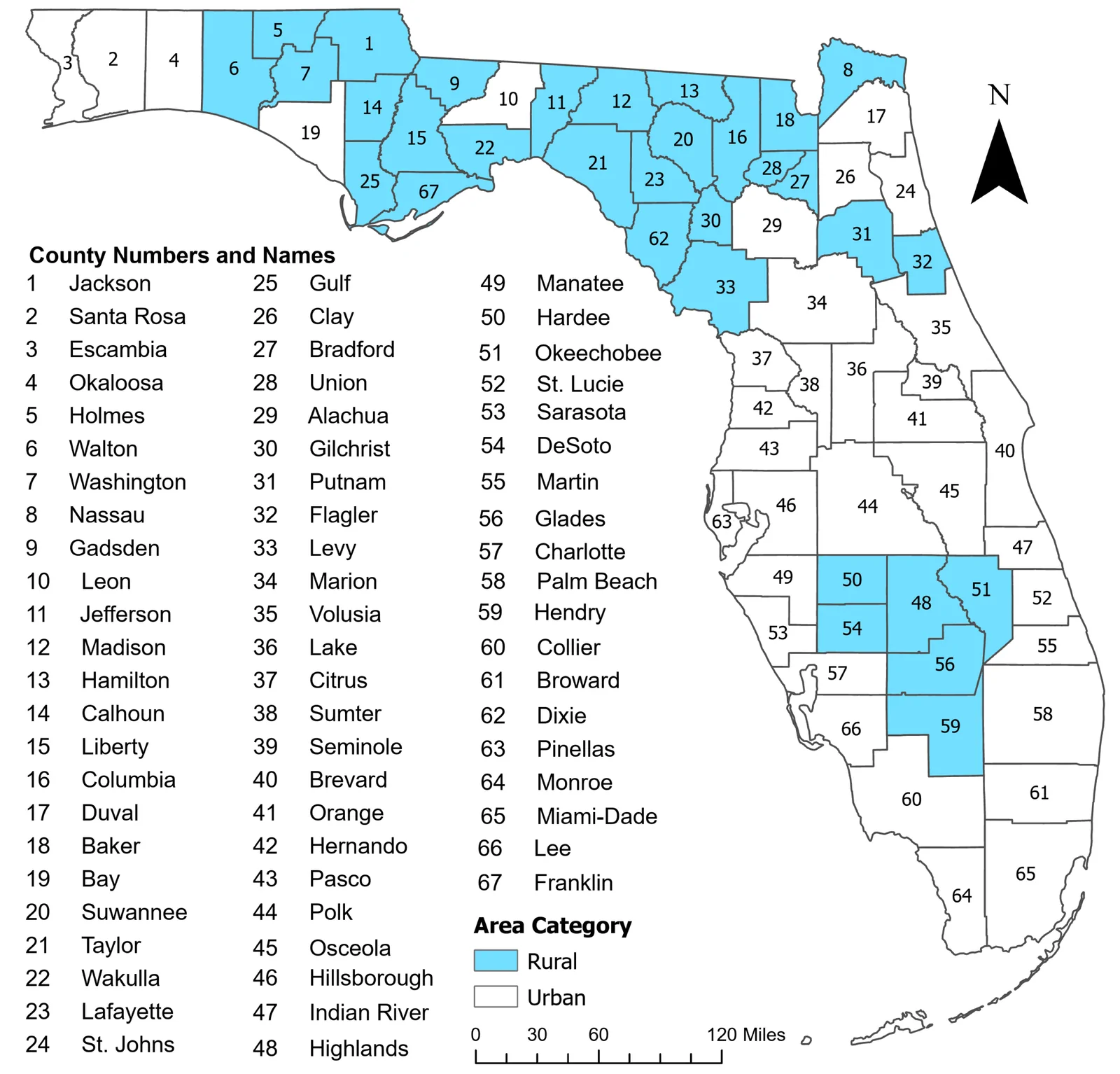
Map of Florida showing county names and the urban/rural classification of the counties. The cartographic boundary file used to create the map was obtained from the United States Census Bureau TIGER Geodatabase. Rural–urban classification for each county was based on the US Department of Commerce 2020 Census classification (United States Department of Commerce, 2020).

### Data sources and management

County-level intentional self-harm hospitalization data based on county of residence for Florida residents for the years 2016–2023, aggregated by age categories: <18 years, 18–34 years, 35–44 years, 45–64 years and ≥ 65 years, were obtained from Florida Health Charts, a publicly available data dashboard maintained by the Florida Department of Health (Florida Department of Health, 2024). It should be noted that the data may contain multiple hospitalizations for the same person or injury event due to hospital transfers, readmissions, and follow-up visits. Therefore, the data reflects the number of hospitalizations and not the number of people who intentionally harmed themselves. The data were split into four 2-year time periods: 2016–2017, 2018–2019, 2020–2021 and 2022–2023. The county-level 5-year population estimates corresponding to the beginning of each analytic period were obtained from the 2016, 2018, 2020, and 2022 American community survey and were used to provide stable county-level denominators for the 2016–2017, 2018–2019, 2020–2021 and 2022–2023 time periods, respectively (American Community Survey, 2025). Given the short duration of each study period (2 years), substantial changes in the population distribution were not expected. The datasets used did not contain missing values for any of the variables used in the analysis. Intentional self-harm hospitalization risk estimates were then calculated and directly age-standardized to the 2010 US population in STATA version 18 (Mehmetoglu & Jakobsen, 2022). The age-standardized hospitalization risks estimates were expressed as the number of hospitalizations per 100,000 persons.

County-level rural–urban classification data used to make rural–urban comparisons of hospitalization risks due to intentional self-harm, were obtained from the US Department of Commerce 2020 Census (United States Department of Commerce, 2020). To account for spatial autocorrelation and variance instability in low-population areas, spatial empirical Bayesian (SEB) smoothed risks were calculated in GeoDa version 1.22.0.2 (Anselin, 2024) using first-order queen contiguity spatial weights (Anselin, Syabri & Kho, 2009; Anselin, 2024). Specifically, smoothing was done based on the following criteria; (i) if the numbers of events that formed the numerator of one or more of the risks were less than three, or (ii) if population at risk per county was small (*e.g.*, <100 or 500) and the numbers changed by an order of magnitude, for instance 10 in some counties and ≥ 100 in others (Leyland & Davies, 2005; Pfeiffer et al., 2008; Anselin, Syabri & Kho, 2009). Cartographic boundary files were obtained from the United States Census Bureau TIGER Geodatabase and used for county-level spatial displays (United States Census Bureau, 2026).

### Investigation of high-risk clusters

Statistically significant clusters of intentional self-harm hospitalization risks were detected using Tango’s Flexible Spatial Scan Statistics (FSSS) (Tango & Takahashi, 2005; Tango & Takahashi, 2012) implemented in FleXScan version 3.1.2 (Takahashi, Yokoyama & Tango, 2010). To avoid the detection of excessively large clusters, the maximum spatial scanning window was set at 15 counties (Tango & Takahashi, 2012). Poisson probability model with a default alpha value of 0.2 (Tango & Takahashi, 2012) and a restricted log likelihood ratio (LLR) were used to ensure that counties with non-elevated risks were not included in high-risk clusters (Tango & Takahashi, 2005; Tango & Takahashi, 2012). For significance testing, 999 Monte Carlo replications and a critical *p*-value of 0.05 were used (Tango & Takahashi, 2012). To avoid reporting low risk clusters, only those with relative risks ≥1.20 were reported. The spatial distribution of the clusters was then mapped and compared visually for each of the time periods 2016–2017, 2018–2019, 2020–2021 and 2022–2023. To further evaluate changes in the spatial distribution of intentional self-harm hospitalization risks, counties identified within statistically significant high-risk clusters across study periods were assessed for persistent (sustained elevated hospitalization risks) and emerging clusters. Persistent high-risk cluster counties were defined as counties that were consistently identified as part of statistically significant high-risk clusters throughout the study periods (2016–2017, 2018–2019, 2020–2021 and 2022–2023). Emerging high-risk cluster counties were defined as counties that were identified as part of statistically significant high-risk clusters during the 2022–2023 period only and were not part of any high-risk clusters during the earlier study periods (2016–2017, 2018–2019, and 2020–2021).

Two sample tests of proportions, implemented in STATA version 18 (Mehmetoglu & Jakobsen, 2022), were used to compare rural–urban counties with respect to the proportion of counties classified as high-risk and the proportion of population residing in high-risk counties. Statistical significance was assessed using two-sided tests at a critical *p*-value of 0.05 (Mehmetoglu & Jakobsen, 2022).

### Cartographic displays

All map visualizations were performed in ArcGISPro version 3.2.0 (Environmental Systems Research Institute, 2024). County-level age-standardized and SEB smoothed hospitalization risks due to intentional self-harm were displayed as choropleth maps using Jenk’s classification scheme (Brewer & Pickle, 2002). Identified high risk clusters of hospitalization risks due to intentional self-harm, including clusters that persisted throughout the study period (2016–2023), and those that emerged during 2022–2023 but were not classified as part of high-risk clusters in earlier time periods (2016–2021), were also mapped in ArcGISPro version 3.2.0 (Environmental Systems Research Institute, 2024).

## Results

### Geographic distribution of intentional self-harm hospitalization risks

A total of 66,308 intentional self-harm hospitalizations were reported during the study period. There was a decrease in the overall intentional self-harm age-standardized hospitalization risk from 93.3 hospitalizations per 100,000 persons in 2016–2017, to 84.2 in 2018–2019, 74.7 in 2020–2021, and 72.9 in 2022–2023. Although, the spatial patterns of the age-standardized risks were similar to those of the SEB smoothed risks, the spatial patterns of the latter were more well defined and identifiable (Figs. 2 and 3). The county-level SEB smoothed risks varied geographically ranging from 25.3 to 1,683.1 hospitalizations per 100,000 persons. The highest risks tended to occur in the northwest, northeast, central, and southwestern parts of the state in 2016–2017 and 2018–2019 (Fig. 3). In 2020–2021 high-risk areas were confided in parts of northwest and northeast Florida. However, in 2022–2023, high-risk areas extended into parts of the northwest, northeast, southwest and upper southeast Florida (Fig. 3).

**Figure 2:**
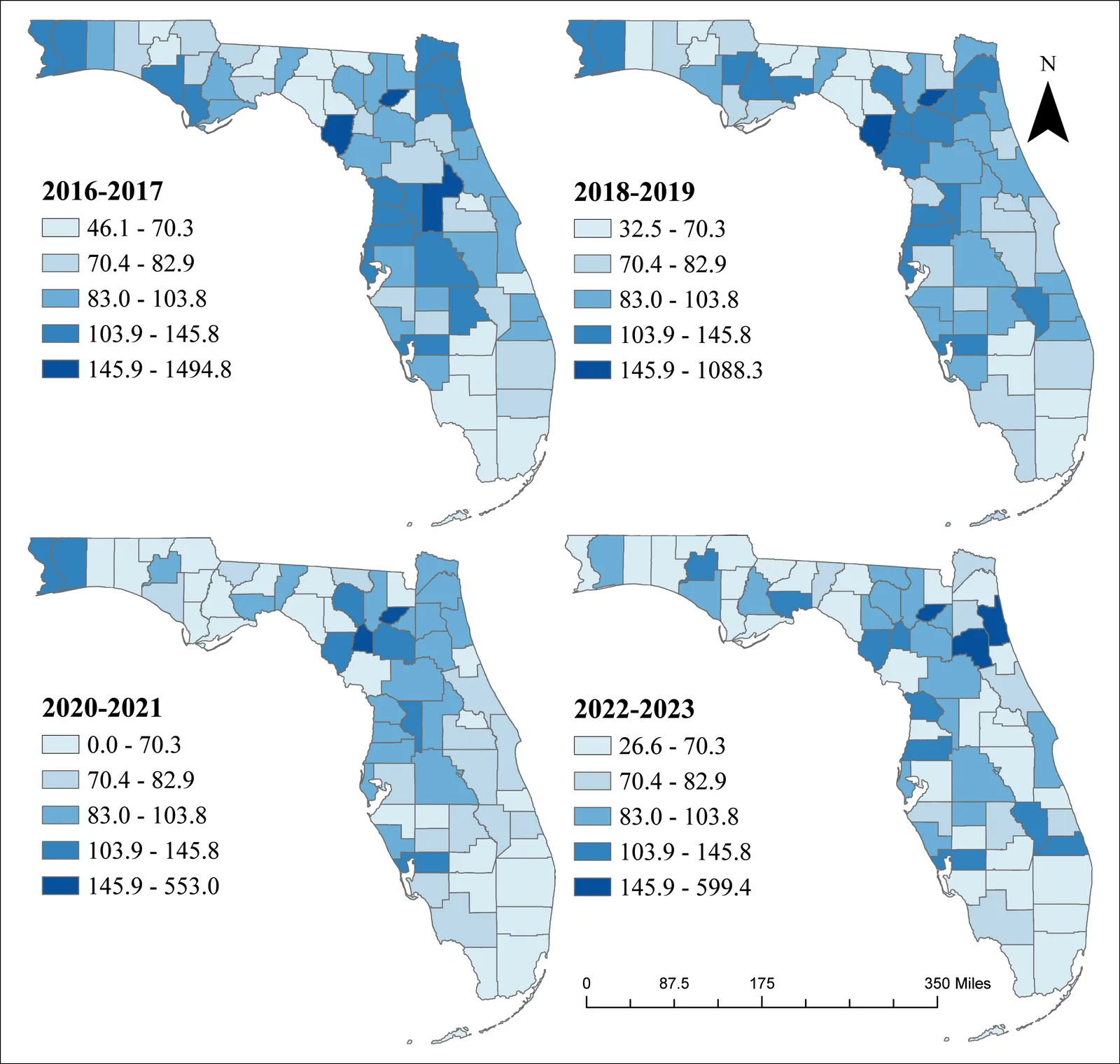
Age-standardized intentional self-harm hospitalization risks in Florida, 2016–2023. The cartographic boundary files were obtained from the United States Census Bureau TIGER Geodatabase. Data displayed were obtained from the Florida Department of Health.

**Figure 3:**
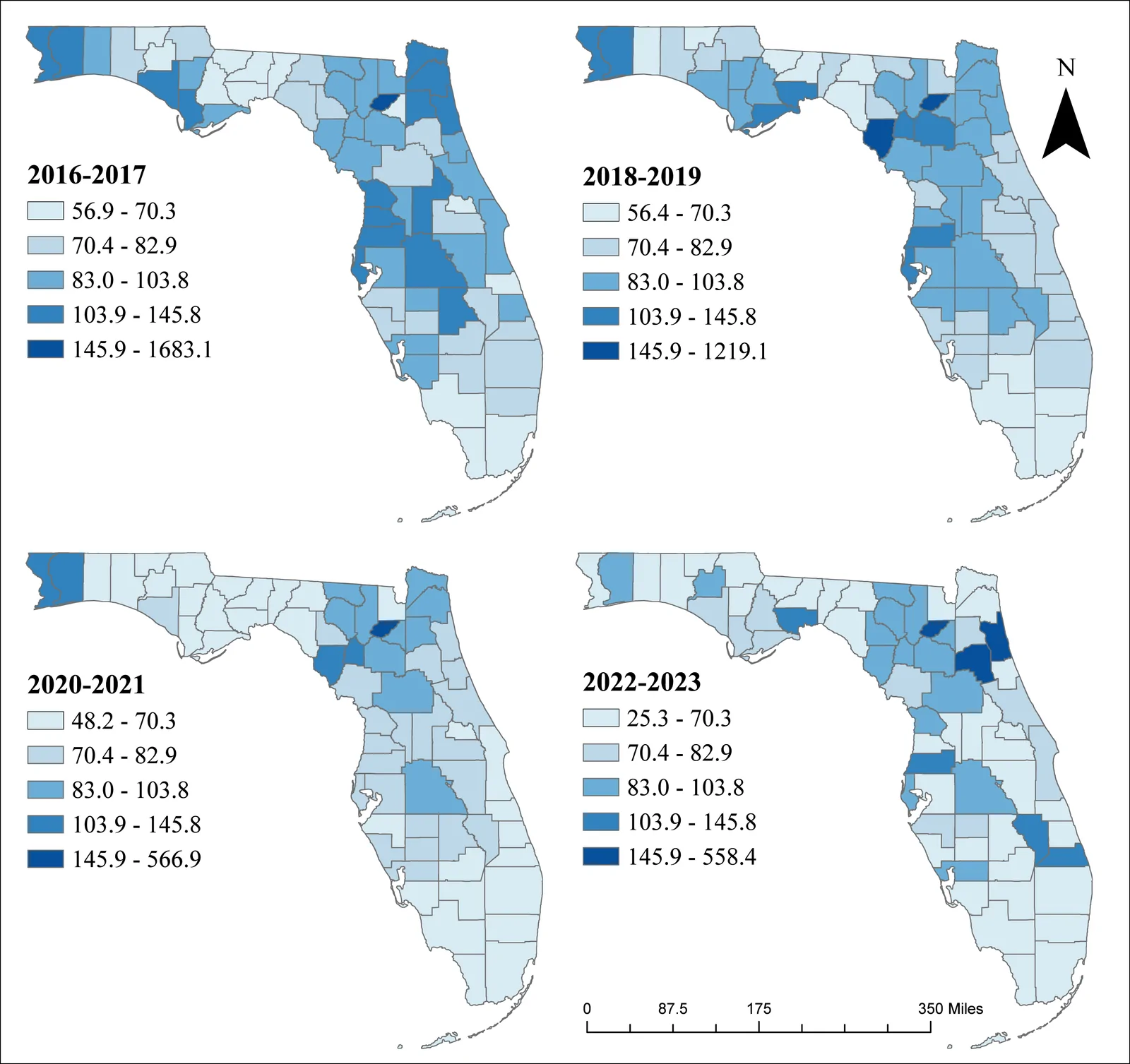
Spatial empirical Bayes (SEB) smoothed intentional self-harm hospitalization risks in Florida, 2016–2023. The cartographic boundary files were obtained from the United States Census Bureau TIGER Geodatabase. Data displayed were obtained from the Florida Department of Health.

Relatively high, more widespread, and stable risks were observed in urban counties located in the northeast including counties of Duval in 2016–2017 (145.8 per 100,000 persons), and St. Johns in 2022–2023 (168.8 per 100,000 persons) (Figs. 1 and 3). In Northwest Florida, Escambia County had relatively high risks among urban counties in 2018–2019 (126.2 per 100,000 persons) and in 2020–2021 (117.9 per 100,000 persons) (Figs. 1 and 3).

### High-risk spatial clusters

#### Geographic distribution and relative risks

Statistically significant clusters of high intentional self-harm hospitalization risks were detected in the northwest, northeast, central, south and southwest parts of Florida (Fig. 4). During 2016–2017 period, four statistically significant clusters were detected, with relative risks (RRs) ranging from 1.44 to 15.85 (Table 1 and Fig. 4). The cluster with the highest relative risk (Cluster 1; RR = 15.85) was a single rural northeastern county (Union) (Figs. 1 and 4). Additional clusters were observed in mainly urban counties in the central (*i.e.,* Lake and Sumter), northwest (*i.e.,* Santa Rosa and Escambia), southwest (*i.e.,* Citrus, Hernando, Pasco, Polk, and Pinellas), and northeastern parts of the state (*i.e.,* Duval, Clay and St. Johns) (Figs. 1 and 4).

**Figure 4:**
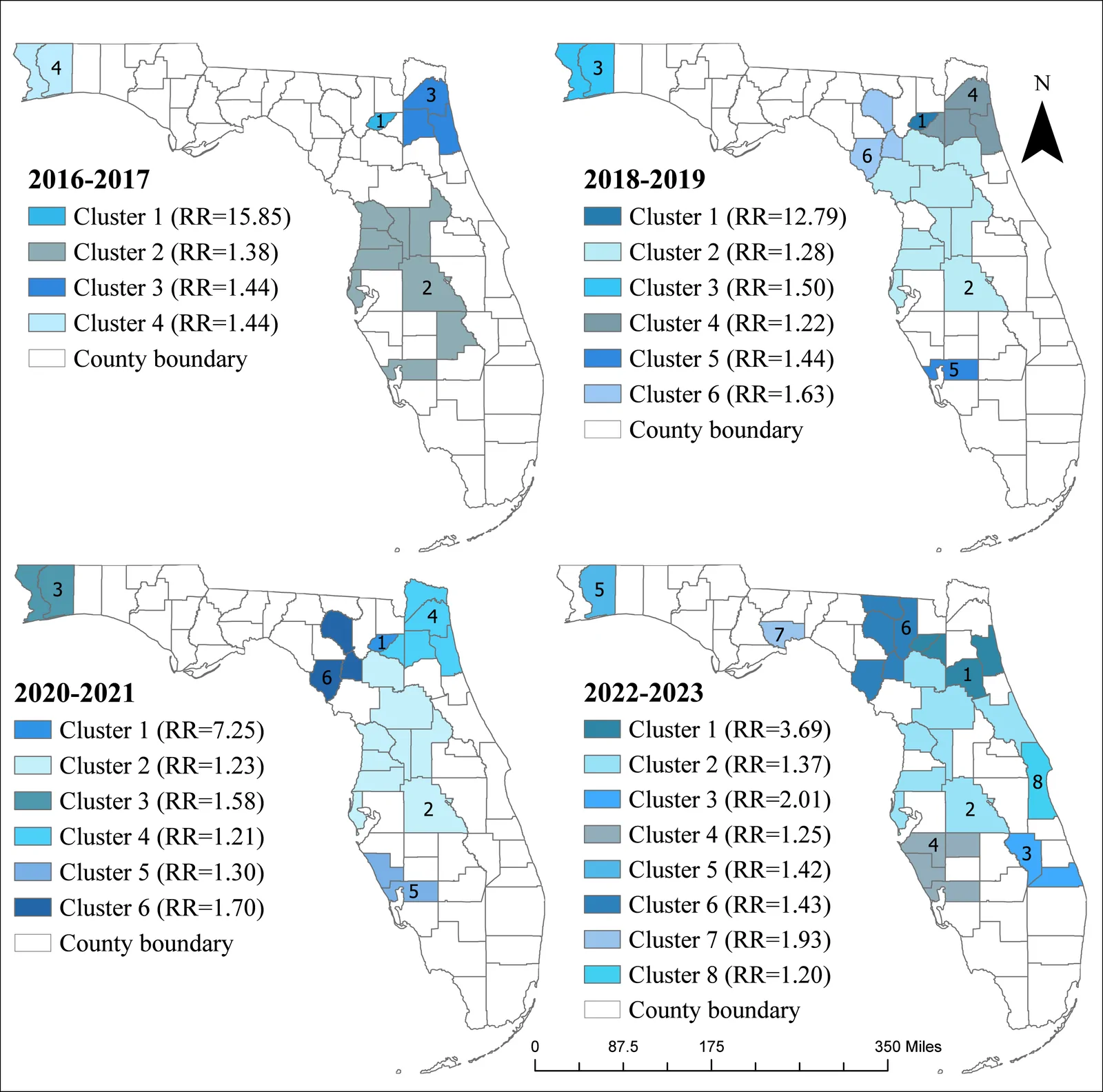
Geographic distribution of spatial clusters of high intentional self-harm hospitalization risks in Florida, 2016–2023. The map was created by the authors using publicly available cartographic boundary files from US Census Bureau TIGER Geodatabase. The data displayed was downloaded from the Florida Department of Health Health Charts Website.

**Table 1:** Spatial clusters of high intentional self-harm hospitalization risks in Florida, 2016–2023.

**Period**	**Cluster**	**Observed cases**	**Expected cases**	**No. counties**	**RR** ^a^	***P*-value**
2016–2017	1	229	14	1	15.85	0.001
	2	4,092	2,962	9	1.38	0.001
	3	1,823	1,267	3	1.44	0.001
	4	649	450	2	1.44	0.001
2018–2019	1	167	13	1	12.79	0.001
	2	3,902	3,041	10	1.28	0.001
	3	624	416	2	1.50	0.001
	4	1,483	1,211	4	1.22	0.001
	5	222	154	1	1.44	0.002
	6	109	67	3	1.63	0.006
2020–2021	1	89	12	1	7.25	0.001
	2	3,461	2,824	9	1.23	0.001
	3	604	382	2	1.58	0.001
	4	1,458	1,202	5	1.21	0.001
	5	609	467	2	1.30	0.001
	6	101	59	3	1.70	0.001
2022–2023	1	1,062	288	4	3.69	0.001
	2	3,700	2,701	8	1.37	0.001
	3	284	141	2	2.01	0.001
	4	957	763	4	1.25	0.001
	5	193	136	1	1.42	0.003
	6	165	115	5	1.43	0.009
	7	47	24	1	1.93	0.019
	8	520	436	1	1.19	0.027

**Notes.**

a Relative risk.

During the 2018–2019 period, there was an increase in the number of statistically significant spatial clusters to six with relative risks ranging from 1.22 to 12.79. The cluster with the highest relative risk (cluster 1; RR = 12.79) was again confined to a single rural northeastern county (Union), yet all the surrounding high-risk clusters were mainly urban counties (Figs. 1 and 4). This period marked the expansion and redistribution of high-risk clusters to predominantly urban counties in the northwest (*i.e.,* Jackson and Santa Rosa), northeast (*i.e.,* Duval, St. Johns, Clay, and Alachua), central (*i.e.,* Marion, Lake, and Sumter), southwest (*i.e.,* Hernando, Pasco, Pinellas, and Polk) and southern part of the state (*i.e.,* Charlotte) (Figs. 1 and 4).

During the 2020–2021 period, the number of statistically significant clusters remained the same at six, with relative risks ranging from 1.21 to 7.25. The cluster with the highest relative risk (Cluster 1; RR = 7.25) again included only a rural county (Union) (Figs. 1 and 4). However, the other five high risk clusters (RRs = 1.21–1.70) were made up of predominantly urban counties. During this period, the identified high-risk urban counties were located in the northwest (*i.e.,* Santa Rosa and Escambia), northeast (*i.e.,* Duval, St. Johns, Clay, and Alachua), central (*i.e.,* Marion, Sumter, and Lake), southwest (*i.e.,* Citrus, Hernando, Pasco, Pinellas, Manatee, and Polk), and southern part of Florida (*i.e.,* Charlotte) (Figs. 1 and 4).

Finally, during the 2022–2023 period, there was an increase in the geographic spread with the number of statistically significant clusters increasing to eight with relative risks ranging from 1.20 to 3.69. During this time, the high-risk clusters were primarily located in urban counties of the northwest (*i.e.,* Santa Rosa), northeast (*i.e.,* St. Johns, and Alachua), central (*i.e.,* Volusia, Brevard, Marion, and Sumter), southwest (*i.e.,* Citrus, Pasco, Pinellas, and Polk), south (*i.e.,* Manatee, Sarasota, Charlotte) and southeast (*i.e.,* Martin) (Figs. 1 and 4).

#### Counties in high-risk clusters

The geographic spread of significant spatial clusters increased over the study period, reaching its peak during the 2022–2023 period. Overall, the proportion of Florida counties included in significant high-risk clusters ranged from 22.4% (15/67) in 2016–2017 to 38.8% (26/67) in 2022–2023 (Table 2). During the 2016–2017 period, the proportion of urban counties (37.1%; 13/67) that were part of high-risk clusters was significantly (*p* = 0.002) higher than that of rural counties (6.3%; 2/32). No significant rural–urban differences were observed during the 2018–2019 period. However, during the 2020–2021 period, significantly (*p* = 0.019) more urban (45.7%; 16/35) than rural (18.8%; 6/32) counties were part of high-risk clusters (Table 2).

**Table 2:** Rural–urban disparities in county level intentional self-harm hospitalization risks in Florida, 2016–2023.

**Clusters**	**Florida (*n* = 67)**			**Rural counties (*n* = 32)**			**Urban counties (*n* = 35)**			*P*-value^c^
	**No** ^a^	**%**	**95% CI** ^b^	**No** ^a^	**%**	**95% CI** ^b^	**No** ^a^	**%**	**95% CI** ^b^	
2016–2017	15	22.4	13.1–34.2	2	6.3	0.8–20.8	13	37.1	21.5–55.1	0.002
2018–2019	21	31.3	20.6–43.8	7	21.9	0.9–40.0	14	40.0	23.9–57.9	0.11
2020–2021	22	32.8	21.8–45.4	6	18.8	7.2–36.4	16	45.7	28.8–63.4	0.019
2022–2023	26	38.8	27.1–51.5	11	34.4	18.6–53.2	15	42.9	26.3–60.6	0.477
Persistent clusters	8	11.9	5.3–22.2	1	3.1	0.0–16.2	7	20.0	8.4–36.9	0.056^*^
Emerging clusters	9	13.4	6.3–24.0	5	15.6	5.3–32.8	4	11.4	3.2–26.7	0.615

**Notes.**

a Number of counties within clusters.

b Binomial exact confidence intervals.

c *P*-value of two sample tests of proportions comparing proportions of rural vs urban counties included in clusters.

* 2-sided Fisher’s exact test.

Urban counties persistently formed parts of high-risk clusters during the first three time periods (2016–2017, 2018–2019, and 2020–2021), continued to appear in all four time periods, or emerged in high-risk clusters during the final period (2022–2023). A total of 11.9% (8/67) of the counties were persistently part of high-risk clusters over the entire study period with no significant (*p* = 0.056) difference between urban (20.0%; 7/35) and rural (3.1%; 1/32) counties (Table 2). The urban counties that persisted in high-risk spatial clusters throughout the study period included: Santa Rosa in the northwest, St. Johns in the northeast, Sumter in the central, Pasco, Pinellas, and Polk in the southwest, and Charlotte in the southern part of Florida (Figs. 1 and 5). Counties that were persistently in high-risk clusters during only the first three time periods (2016–2017, 2018–2019, and 2020–2021) were in the northwest (*i.e.,* Escambia and Santa Rosa), northeast (*i.e.,* Duval, St. Johns, Clay), central (*i.e.,* Lake and Sumter), southwest (*i.e.,* Hernando, Pasco, Pinellas, Polk) and south (*i.e.,* Charlotte).

**Figure 5:**
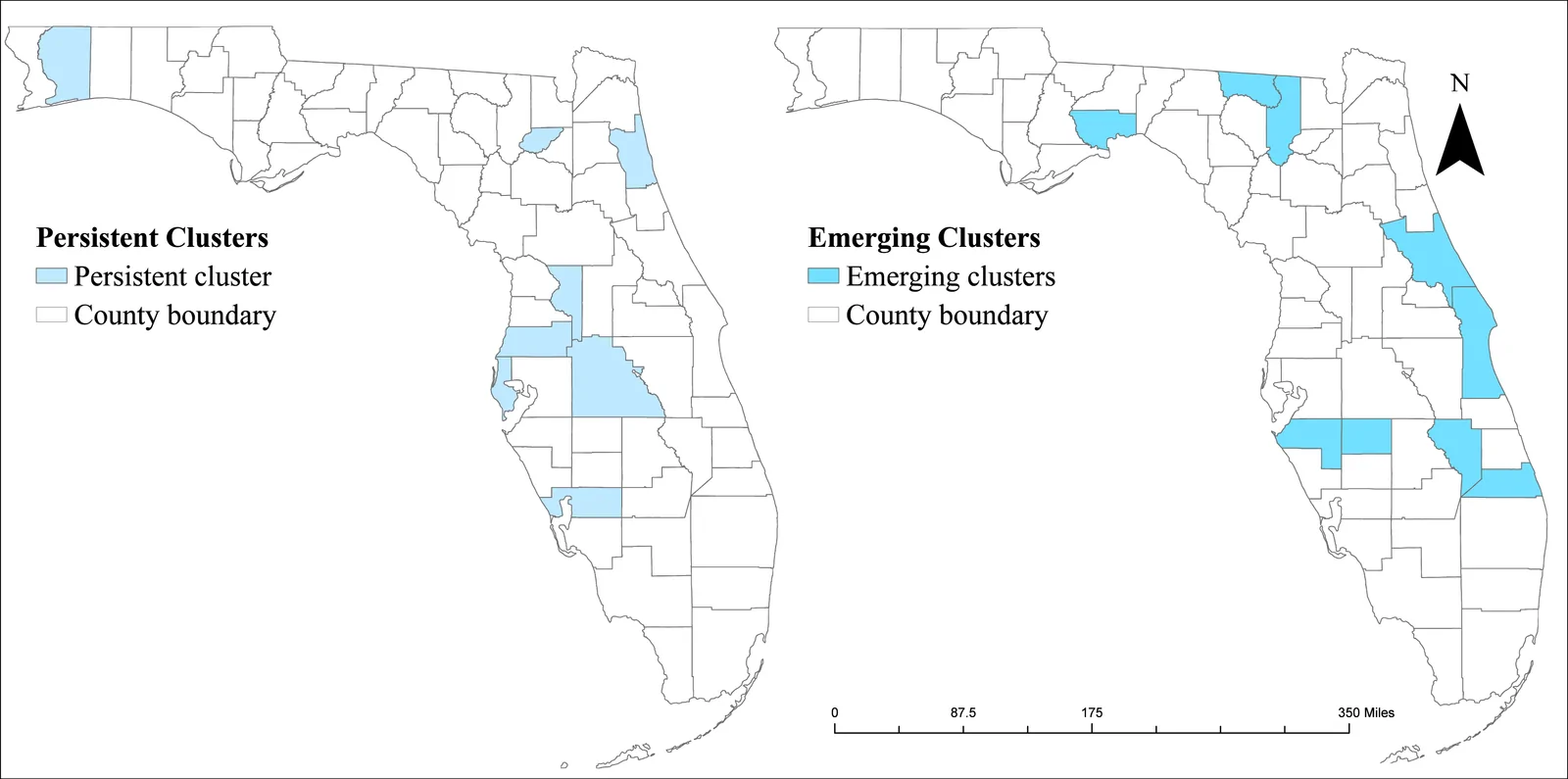
Geographic distribution of spatial clusters of persistent and emerging high intentional self-harm hospitalization risks in Florida, 2016–2023. The map was created by the authors using publicly available cartographic boundary files from US Census Bureau TIGER Geodatabase. The data displayed was downloaded form the Florida Department of Health Health Charts Website.

#### Populations in high-risk clusters

As many as 2.9 million Florida residents lived in counties that were persistently in high-risk clusters over the study period. The proportion of the population residing in high-risk cluster counties increased from 24.5% in 2016–2017 to 30.5% in 2020–2021, then slightly decreased to 29.9% in 2022–2023. The proportion of urban population that lived in persistently high-risk counties throughout the study period (14.7%) was significantly (*p* < 0.0001) higher than that in rural counties (1.3%) (Table 3).

**Table 3:** Rural–urban population distribution in high-risk clusters of hospitalization due to intentional self-harm.

**Clusters**	**Florida population**			**Rural population**			**Urban population**			*P*-value^j^
	**TP** ^a^	**CC** ^b^	**%** ^c^	**RC** ^d^	**RCC** ^e^	**%** ^f^	**UC** ^g^	**UCC** ^h^	**%** ^i^	
2016–2017	20,278,447	4,971,047	24.5	1,128,744	115,477	10.2	19,149,703	4,855,570	25.4	<0.0001
2018–2019	20,901,636	6,306,310	30.2	1,155,021	793,053	68.7	19,746,615	5,513,257	27.9	<0.0001
2020–2021	21,339,762	6,508,754	30.5	1,158,650	210,535	18.2	20,181,112	6,298,219	31.2	<0.0001
2022–2023	21,928,881	6,553,432	29.9	1,189,384	382,169	32.1	20,739,497	6,171,263	29.8	<0.0001
Persistent clusters	20,901,636	2,916,329	14.0	1,155,021	15,303	1.3	19,746,615	2,901,026	14.7	<0.0001
Emerging clusters	21,928,881	1,949,811	8.9	1,189,384	184,565	15.5	20,739,497	1,765,246	8.5	<0.0001

**Notes.**

a TP, Total population of Florida.

b CC, Population living in high-risk cluster counties.

c Percentage of population living in high-risk cluster counties (CC/TP).

d RC, Population living in rural counties.

e RCC, Population in rural counties that were part of a high-risk cluster.

f % Percentage of rural population living in high-risk cluster counties (RCC/RC).

g UC, Population living in urban counties.

h UCC, Population in urban counties that were part of a high-risk cluster.

i % Percentage of urban population living in high-risk cluster counties (UCC/UC).

j *P*-value of two sample test of proportions comparing rural and urban counties.

It is worth noting that intentional self-harm hospitalization risks in Florida showed a dynamic rural–urban pattern that changed over time. For instance, during the 2016–2017 time period, the proportion of urban populations living in high-risk cluster counties (25.4%) was significantly (*p* < 0.0001) higher than that in rural counties (10.2%) (Table 3). However, during the 2018–2019 period the disparity shifted to rural populations where significantly (*p* < 0.0001) more rural (68.7%) than urban (27.9%) populations lived in high-risk cluster counties. During the 2020–2021 period this shifted to urban populations, where the proportion of urban population in high-risk counties (31.2%) was significantly (*p* < 0.0001) higher than that in rural counties (18.2%) (Table 3). Finally, during the 2022–2023 period, again significantly (*p* < 0.0001) more rural (32.1%) than urban (29.8%) populations resided in counties that were part of high-risk clusters. Additionally, during the same period, the proportion of rural population in high-risk counties that were part of emerging clusters (15.5%) was significantly (*p* < 0.0001) higher than that in urban counties (8.5%) (Table 3).

Overall, these results show that the geographic distribution of intentional self-harm hospitalization risks in Florida is not static but changes over time between urban and rural populations. Although urban counties consistently account for the largest share of population living in high-risk cluster counties, rural populations experience episodic surges in intentional self-harm hospitalization risks. Additionally, rural populations tended to reside in counties that were part of newly emerging high-risk clusters.

## Discussion

This study investigated and identified county-level geographic disparities of intentional self-harm hospitalization risks in Florida. Although the statewide age-standardized and SEB smoothed intentional self-harm hospitalization risks decreased over the study period, high risks were observed in predominantly urban counties. Study findings are useful for guiding the allocation of resources to high-risk areas with the aim of reducing/eliminating disparities and improving mental health outcomes for all.

### Geographic distribution

The disparities in intentional self-harm hospitalization risks observed across both urban and rural counties in the northwest, northeast, central, and southwestern parts of Florida are consistent with reports from other studies that reported that intentional self-harm hospitalization risks remain a significant concern. Disparities have been reported in the United States (urban areas in the south (Oladunjoye et al., 2020), and throughout rural America (Arakelyan, Emond & Leyenaar, 2022)), India (rural areas in the northeast (Nongpiur & Tesia, 2018) and south India (Hosahally & Raj, 2024)), all Canadian provinces, except Quebec, (Liu et al., 2024), England (urban northwest, East midlands, and Southeast parts (Clements et al., 2016; Geulayov et al., 2018)), Sweden (urban east-central regions (Lodebo et al., 2017)), and Uganda (rural northern (Kaggwa et al., 2024), eastern (Pedersen et al., 2017) and urban central and western regions (Pedersen et al., 2017; Favina, Abaatyo & Kaggwa, 2024)). The reason for the high hospitalization risks in urban areas is consistent with the Central Place Theory, which hypothesizes that higher-order healthcare services such as inpatient care for intentional self-harm are geographically concentrated in urban centers (Xiang et al., 2025). This theory is consistent with the current study’s finding that showed that while rural counties in Florida experienced periodic spikes in intentional self-harm hospitalization risks, urban counties had more consistent widespread high hospitalization risks (Saunders et al., 2025).

Interestingly, the highest hospitalization risks were observed in the rural northeastern county of Union, potentially reflecting limited access to mental health care in these areas, which may delay detection and management of self-harm, increasing the likelihood of hospitalizations (Florida Department of Health, 2020). The substantially higher population-to-mental-health-provider ratio in Union County (1,726:1) compared to the Florida state average (459:1) indicates a severe shortage of mental health services at the local level (County Health Rankings & Roadmaps, 2026). Florida Psychological Association reported that 86.6% of Florida counties lacked psychiatrists (Florida Psychological Association, 2025), compared to 65% of rural counties nationwide (Andrilla et al., 2018). It is worth noting that as of 2025, there were no psychiatrists in Union County (Florida Psychological Association, 2025). Limited mental health provider availability likely delays diagnosis and treatment, increasing risks of hospitalizations (Rural Health Information Hub, 2025). This shows systemic vulnerabilities in access to healthcare in rural areas and the need for targeted health planning and resource allocation to prevent poor self-harm outcomes in resource limited settings (Harriss & Hawton, 2011; Friedman & Holmes, 2022). It should be noted that provider availability was not directly measured as a covariate in the current analysis. As such, this factor is intended to provide a broader contextual interpretation of the observed spatial and rural–urban patterns rather than imply statistically modeled relationships. This should therefore be interpreted as plausible, hypothesis explanations drawn from existing literature.

### High-risk spatial clusters

#### Geographic distribution and relative risks

The high intentional self-harm hospitalization risks observed in urban counties reflect utilization patterns of health services driven by greater service availability rather than a higher burden of the problem in urban counties (Andrilla et al., 2018; Nuako et al., 2022; Shrader et al., 2023). For instance, a study in Florida showed a high density of behavioral health treatment facilities in urban counties of northwest, northeast, central, southwest, and southeast parts of the state (Shrader et al., 2023). The major metropolitan statistical areas including Miami-Fort Lauderdale-Palm Beach in the southeast, Tampa-St Petersburg-Clearwater in the southwest, Orlando-Kissimmee-Sandford in the central, Jacksonville in the northeast, Tallahassee and Pensacola-Ferry Pass Brent in the northwest parts of Florida, serve as regional hubs (Shrader et al., 2023). This likely amplifies disparities in healthcare utilization by channeling high-acuity care such as severe intentional self-harm toward metropolitan areas (Nuako et al., 2022).

#### Counties and populations in high-risk clusters

The observed increasing proportion of Florida counties and populations in high-risk clusters, alongside shifts in cluster composition between urban and rural counties, suggests that evolving spatial patterns of self-harm hospitalization risks are partly driven by socioeconomic deprivation (Polling et al., 2019; Edwards et al., 2022; Satherley et al., 2022; Liu et al., 2024). A population-based study of all Canadian provinces, except Quebec, involving 109,398 self-harm hospitalizations found substantially higher hospitalizations in materially and socially deprived areas, with residents of the most materially deprived areas experiencing approximately 50% higher hospitalizations and those in the most socially deprived areas at 70% compared with residents of the least deprived areas (Liu et al., 2024). However, that study did not stratify their analyses by rural–urban designation. A 22-year Danish study reported that urban residents of low socioeconomic position residing in deprived neighborhoods had higher risks of self-harm compared with equivalently disadvantaged urban residents residing in affluent areas (Ejlskov et al., 2023). These reports were similar to those of Lodebo et al. (2017) in Stockholm, Sweden. However, studies conducted in rural and urban districts of Oxfordshire, England and urban South East London reported substantially higher risks of intentional self-harm in urban compared to rural areas even after adjusting for socioeconomic deprivation and social fragmentation, suggesting that high rural hospitalization risks may be influenced by local contextual and health system factors beyond social deprivation alone (Harriss & Hawton, 2011; Polling et al., 2019). It should be noted that socioeconomic conditions were not directly assessed in this study. Similarly, cross national comparisons should be interpreted cautiously, as differences in healthcare systems, surveillance practices, socioeconomic structures, and behavioral health service availability may limit direct comparability with the US context and may introduce contextual confounding not accounted for in the present analysis.

### Temporal distribution

The observed decline in the age-standardized and SEB smoothed intentional self-harm hospitalization risks in this study is consistent with reports from previous studies that attributed this to under ascertainment rather than true reduction in self-harm cases (Patrick et al., 2010; Ghaderi et al., 2022; Law et al., 2022; Simon et al., 2022; Florida Department of Health, 2026). The Florida Department of Health notes that intentional self-harm hospitalization data for the specific mechanisms of intentional self-injury may be underestimated due to incomplete reporting of the external cause of injury (Florida Department of Health, 2026). The study by Simon and co-workers reported that in seven California hospitals, ICD-10 self-harm codes missed about 10% of intentional self-harm cases among high-risk populations, with these events misclassified in clinical records as accidental, undetermined, or lacking documentation (Simon et al., 2022). In the current study, under ascertainment may be a plausible explanation, at least in part, for the low hospitalization risks observed at the beginning of the study period. On the other hand, Jollant et al. (2021), Ghaderi et al. (2022), and Law et al. (2022) attributed the decrease to the COVID19 confinement period characterized by self-isolation and stay at home orders. Taken together, these suggest that the reported intentional self-harm hospitalization counts may underestimate the true intentional self-harm hospitalization risks.

### Practical implications of observed disparities

The identification of county-level geographic disparities, high risk clusters, and rural urban differences in intentional self-harm hospitalization risks has important implications for geographically targeted prevention and behavioral health resource allocation (Rivera-González et al., 2022; Becker-Haimes et al., 2024; Ku et al., 2025). In Florida, state and federal partners, including the Florida Department of Children and Families (Florida Department of Children and Families, 2024), and the Substance Abuse and Mental Health Services Administration (Substance Abuse and Mental Health Services Administration, 2023), support a range of behavioral health resources. These include crisis stabilization services, mobile response teams, suicide prevention lifelines (*e.g.*, 988 crisis services), community mental health centers, certified community behavioral health clinics, and federally funded grants for suicide and intentional self-harm prevention, and rural behavioral health expansion (Wishon & Brown, 2023; Becker-Haimes et al., 2024). These resources may be particularly relevant for counties identified as part of high-risk clusters with the highest relative risks of intentional self-harm hospitalizations, particularly those observed in the northeast, central, south and southwest parts of the state. Since the passage of the Patient Protection and Affordable Care Act in 2014, states had the option to extend Medicaid eligibility, the US’s largest funding of mental health, to individuals ages 18–64 with incomes below 138% of the federal poverty level ($20,783 for an individual in 2024) (Ku et al., 2025). However, Florida has not adopted Medicaid expansion, leaving many low-income households uninsured despite earning too much to qualify for traditional Medicaid and too little to receive marketplace subsidy assistance (Rivera-González et al., 2022). This coverage gap has important implications for access to behavioral healthcare, particularly in geographically underserved counties with elevated self-harm hospitalization risks (Rivera-González et al., 2022; Ku et al., 2025). Although the Florida Medicaid Managed Care Program currently supports specialized therapeutic foster care services, statewide inpatient psychiatric program services, and therapeutic behavioral onsite services for covered populations (State of Florida Agency for Health Care Administration, 2023), many vulnerable adults remain outside the coverage systems (National Alliance on Mental Illness, 2025). In 2023, Florida received $23 billion in federal support for Medicaid (National Alliance on Mental Illness, 2025). Therefore, geographically targeted expansion of Medicaid to these high utilization counties based on relative risks of high-risk clusters through prioritizing deployment of mobile crisis services, strengthening local behavioral health workforce capacity, and expanding tele-mental health infrastructure would reflect a data-driven approach to aligning service availability with geographic variation in need (Rivera-González et al., 2022; Wishon & Brown, 2023; Becker-Haimes et al., 2024). This would likely improve access to timely crisis intervention and continuity of care in settings with high utilization of intentional self-harm services (Rivera-González et al., 2022; Becker-Haimes et al., 2024). However, as a county-level ecological analysis, this study cannot distinguish whether observed geographic variation reflects differences in underlying risk, healthcare utilization patterns, coding practices, or a combination of these factors. It should also be noted that although these system level resource allocation mechanisms were not directly evaluated in the current study, the observed spatial heterogeneity in risk of intentional self-harm hospitalizations provides the potential value of integrating epidemiologic cluster detection with prevention planning. These considerations are intended as context for interpreting the findings and as a basis for future research as more actionable intervention planning and resource allocation would benefit from statistically modeling the predictors/determinants of the observed disparities. Future studies by this research team will address these aspects.

### Strengths and limitations

A key strength of this study is the application of rigorous spatial epidemiological techniques to: (1) adjust for spatial autocorrelation and variance instability in risk mapping and (2) identify high-risk clusters and disparities in intentional self-harm hospitalizations risks. In addition, stratification by urban–rural status and assessment across four time periods provided insight into the dynamic nature of intentional self-harm hospitalization risks and the persistence or emergence of high-risk areas. However, this study is not without limitations. The geographic patterns identified may be different from those at lower spatial scales of analysis such as neighborhoods or zip code levels. Unfortunately, the data used for this study was only available at the county level, therefore, analyses could not be done at lower spatial scales. Intentional self-harm hospitalizations may be under ascertained due to incomplete or inaccurate external cause of injury coding, potentially resulting in underestimation of the true utilization of intentional self-harm hospitalizations. Geographic differences in healthcare reporting practices, stigma, healthcare access, and diagnostic classification may contribute to spatial variation in ascertainment across counties. The outcome is visit based and not person-based, so multiple visits by the same person were treated as separate events. This study investigated intentional self-harm as a broad composite outcome and did not evaluate differences by self-harm subtype, or mechanism of injury. Although more targeted stratified analyses may provide additional insight into the geographic distribution of intentional self-harm, hospitalization counts for specific mechanisms of injury within the data source used in this study (including firearm-related injury, drug poisoning, cut/pierce injuries, and other mechanisms), were too low in many of the counties to support spatial analyses. Additionally, this study, which is part of a larger research program investigating the epidemiology of intentional self-harm, was designed to identify geographic disparities in intentional self-harm hospitalization risks and did not investigate predictors/determinants of the identified disparities. Future studies, which are part of the research program, will investigate the underlying predictors/determinants of these disparities. Thus, the analysis did not assess the contribution of potential predictors such as age, sex, race/ethnicity, socioeconomic conditions, mental health burden, healthcare access, access to lethal means (*e.g.*, firearms), area level poverty or deprivation, or other social determinants of health that may be associated with the outcome of the observed spatial disparities because these will be investigated in a separate study. Additionally, since this study did not perform subgroup-specific analyses or adjust for confounding, the findings should be interpreted as a first step in understanding geographical disparities and more information will be gained to get a more complete understanding upon completion of the follow up studies. As mentioned earlier, future studies will investigate determinants of the identified disparities and will provide a more complete picture to inform health programs to address the problem. Although age standardized risks allow meaningful comparisons across counties by accounting for differences in population age-structure, age stratified county level analysis was not statistically feasible due to low county-level age-specific hospitalization counts. Furthermore, the data source used in this study did not provide county-level hospitalization counts stratified by sex, preventing sex-specific analyses despite the known differences in intentional self-harm patterns between males and females. Therefore, high-risk age groups such as adolescents or females may exhibit spatial patterns that differ from those observed in the aggregated estimates used in this study. Although this study applied spatial empirical Bayes smoothing, estimates in sparsely populated counties may remain subject to some degree of instability and it does not account for year-to-year variability in case occurrence. This is particularly relevant for counties with rare intentional self-harm hospitalizations where even small changes in case counts may result in relatively large fluctuations in estimated risks. Therefore, extreme values observed in single-county clusters should be interpreted as signals of potential elevated risk. Despite these limitations, the findings are important for guiding health planning and needs-based allocation of resources geared towards reducing disparities and improving mental health outcomes.

## Conclusion

Despite a statewide decline in intentional self-harm hospitalization risks from 2016 to 2023, this study shows evidence of geographic disparities across Florida, characterized by high-risk clusters in urban counties and episodic surges in rural populations. Targeted interventions are important to reduce/eliminate these disparities and improve outcomes for all. Future research should investigate predictors of the identified disparities and examine whether spatial patterns differ by self-harm subtype, suicidal intent, and mechanism of injury, including firearm related injury, drug poisoning, suffocation, and cut/pierce injuries to better guide intervention efforts.

## Supplemental Information

10.7717/peerj.21783/supp-1 Supplemental Information 1 Data

10.7717/peerj.21783/supp-2 Supplemental Information 2 STROBE checklist

## Additional Information and Declarations

### Data Availability

The following information was supplied regarding data availability: The data is available in the Supplemental Files.
