# Supplementary material for: Geographical disparities in intentional self-harm hospitalization risks in Florida: a retrospective ecological study
Source: PeerJ. 2026 Oct 8;14:e21783. doi: 10.7717/peerj.21783 (PMC13654277; doi:10.7717/peerj.21783)
Supplement: Supplementary file 1 [file peerj-14-21783-s002.doc]

STROBE Statement—Checklist of items that should be included in reports of ***cross-sectional studies***

|  | Item No | Recommendation |
| --- | --- | --- |
| **Title and abstract** | 1 | (*a*) Indicate the study’s design with a commonly used term in the title or the abstract  **Location in Manuscript: Study’s design has been indicated in the title** |
|  |  | (*b*) Provide in the abstract an informative and balanced summary of what was done and what was found  **Location in manuscript: Abstract (Background, methods, results, conclusion)** |
| Introduction | | |
| Background/rationale | 2 | Explain the scientific background and rationale for the investigation being reported  **Location in manuscript: Introduction, paragraphs 1-4** |
| Objectives | 3 | State specific objectives, including any prespecified hypotheses  **Location in manuscript: End of introduction – paragraph 4** |
| Methods | | |
| Study design | 4 | Present key elements of study design early in the paper  **Location in manuscript: Materials and methods (Study design and setting)** |
| Setting | 5 | Describe the setting, locations, and relevant dates, including periods of recruitment, exposure, follow-up, and data collection  **Location in manuscript: Materials and methods (Study design and setting; data sources and management)** |
| Participants | 6 | (*a*) Give the eligibility criteria, and the sources and methods of selection of participants  **Location in manuscript: Not applicable – ecological aggregated county-level data** |
| Variables | 7 | Clearly define all outcomes, exposures, predictors, potential confounders, and effect modifiers. Give diagnostic criteria, if applicable  **Location in manuscript: Materials and methods (Data sources and management)** |
| Data sources/ measurement | 8* | For each variable of interest, give sources of data and details of methods of assessment (measurement). Describe comparability of assessment methods if there is more than one group  **Location in manuscript: Materials and methods (Data sources and management, investigation of high-risk clusters, cartographic displays)** |
| Bias | 9 | Describe any efforts to address potential sources of bias  **Location in manuscript: Strengths and limitations** |
| Study size | 10 | Explain how the study size was arrived at  **Location in manuscript: Not applicable** |
| Quantitative variables | 11 | Explain how quantitative variables were handled in the analyses. If applicable, describe which groupings were chosen and why  **Location in manuscript: Materials and methods (Data sources and management, investigation of high-risk clusters, cartographic displays)** |
| Statistical methods | 12 | (*a*) Describe all statistical methods, including those used to control for confounding  **Location in manuscript: Materials and methods (Data sources and management, investigation of high-risk clusters, cartographic displays)** |
|  |  | (*b*) Describe any methods used to examine subgroups and interactions  **Location in manuscript: Materials and methods (investigation of high-risk clusters)** |
|  |  | (*c*) Explain how missing data were addressed  **Location in manuscript: Materials and methods (Data sources and management)** |
|  |  | (*d*) If applicable, describe analytical methods taking account of sampling strategy  **Location in manuscript: Not applicable** |
|  |  | (*e*) Describe any sensitivity analyses  **Location in manuscript: None performed** |
| Results | | |
| Participants | 13* | (*a*) Report numbers of individuals at each stage of study—eg numbers potentially eligible, examined for eligibility, confirmed eligible, included in the study, completing follow-up, and analysed  **Location in manuscript: Not applicable – no individuals. County level hospitalizations reported** |
|  |  | (b) Give reasons for non-participation at each stage  **Location in manuscript: Not applicable** |
|  |  | (c) Consider use of a flow diagram  **Location in manuscript: Not applicable** |
| Descriptive data | 14* | (*a*) Give characteristics of study participants (eg demographic, clinical, social) and information on exposures and potential confounders  **Location in manuscript: Materials and methods (Data sources and management, investigation of high-risk clusters, cartographic displays)** |
|  |  | (b) Indicate number of participants with missing data for each variable of interest  **Location in manuscript: Not applicable. Missingness discussed in Materials and methods (data sources and management)** |
| Outcome data | 15* | Report numbers of outcome events or summary measures  **Location in manuscript: Results (Geographic distribution of intentional self-harm hospitalization risks)** |
| Main results | 16 | (*a*) Give unadjusted estimates and, if applicable, confounder-adjusted estimates and their precision (eg, 95% confidence interval). Make clear which confounders were adjusted for and why they were included  **Location in manuscript: Results (Tables 2-3)** |
|  |  | (*b*) Report category boundaries when continuous variables were categorized  **Location in manuscript: Results (Geographic distribution of intentional self-harm hospitalization risks, High-risk spatial clusters)** |
|  |  | (*c*) If relevant, consider translating estimates of relative risk into absolute risk for a meaningful time period  **Location in manuscript: High-risk spatial clusters** |
| Other analyses | 17 | Report other analyses done—eg analyses of subgroups and interactions, and sensitivity analyses  **Location in manuscript: Results (High risk spatial clusters)** |
| Discussion | | |
| Key results | 18 | Summarise key results with reference to study objectives  **Location in manuscript: Discussion: Opening paragraph** |
| Limitations | 19 | Discuss limitations of the study, taking into account sources of potential bias or imprecision. Discuss both direction and magnitude of any potential bias  **Location in manuscript: Strengths and limitations section** |
| Interpretation | 20 | Give a cautious overall interpretation of results considering objectives, limitations, multiplicity of analyses, results from similar studies, and other relevant evidence  **Location in manuscript: Discussion paragraphs 1-5** |
| Generalisability | 21 | Discuss the generalisability (external validity) of the study results  **Location in manuscript: Discussion (rural-urban implications)** |
| Other information | | |
| Funding | 22 | Give the source of funding and the role of the funders for the present study and, if applicable, for the original study on which the present article is based  **Location in manuscript: Funding section** |

*Give information separately for exposed and unexposed groups.

**Note:** An Explanation and Elaboration article discusses each checklist item and gives methodological background and published examples of transparent reporting. The STROBE checklist is best used in conjunction with this article (freely available on the Web sites of PLoS Medicine at http://www.plosmedicine.org/, Annals of Internal Medicine at http://www.annals.org/, and Epidemiology at http://www.epidem.com/). Information on the STROBE Initiative is available at www.strobe-statement.org.
